# Immune cell mediated cabozantinib resistance for patients with renal cell carcinoma

**DOI:** 10.1093/intbio/zyab018

**Published:** 2021-12-21

**Authors:** Keon Young Park, Hunter O Hefti, Peng Liu, Karina M Lugo-Cintrón, Sheena C Kerr, David J Beebe

**Affiliations:** 1 Department of Surgery, University of California San Francisco, San Francisco, CA, USA; 2 Department of Biomedical Engineering, University of Wisconsin, Madison, WI, USA; 3 Department of Biostatistics and Medical Informatics, University of Wisconsin School of Medicine and Public Health, Madison, WI, USA; 4 Carbone Cancer Center, University of Wisconsin, Madison, WI, USA; 5 Department of Pathology and Laboratory Medicine, University of Wisconsin School of Medicine and Public Health, Madison, WI, USA

**Keywords:** renal cell carcinoma, cabozantinib, microfluidics, tumor microenvironment, cancer immunology

## Abstract

Renal cell carcinoma (RCC) is the third most common genitourinary cancer in the USA. Despite recent advances in the treatment for advanced and metastatic clear cell RCC (ccRCC), the 5-year relative survival rate for the distant disease remains at 12%. Cabozantinib, a tyrosine kinase inhibitor (TKI), which is one of the first-line therapies approved to treat advanced ccRCC as a single agent, is now being investigated as a combination therapy with newer immunotherapeutic agents. However, not much is known about how cabozantinib modulates the immune system. Here, we present a high throughput tri-culture model that incorporates cancer cells, endothelial cells, and patient-derived immune cells to study the effect of immune cells from patients with ccRCC on angiogenesis and cabozantinib resistance. We show that circulating immune cells from patients with ccRCC induce cabozantinib resistance via increased secretion of a set of pro-angiogenic factors. Using multivariate partial least square regression modeling, we identified CD4+ T cell subsets that are correlated with cabozantinib resistance and report the changes in the frequency of these populations in ccRCC patients who are undergoing cabozantinib therapy. These findings provide a potential set of biomarkers that should be further investigated in the current TKI-immunotherapy combination clinical trials to improve personalized treatments for patients with ccRCC.

**Insight Box**

Despite the advances in treatment for advanced renal cell carcinoma (RCC), the survival rate remains low. Therefore, many clinical trials are investigating the efficacy of combined tyrosine kinase inhibitors (TKIs) therapy and immunotherapies. However, not much is known about how cabozantinib, one of the clinically approved first-line TKIs to treat advanced RCC, modulates the immune system, leading to treatment resistance. Here, using a high throughput multi-culture system, we show that immune cells from patients with RCC induce cabozantinib resistance via increased secretion of certain pro-angiogenic factors. We also identify T cell subsets that are correlated with the resistance. In addition, this multi-culture system is readily usable in conventional biomedical laboratories with different cell types, and the small culture size allows the use of cells from patients.

## INTRODUCTION

Renal cell carcinoma (RCC) is the third most common genitourinary cancer in the USA. Clear cell renal cell carcinoma (ccRCC) is the most common subtype and accounts for up to 85% of RCC. Despite recent advances in treatment for advanced and metastatic RCC [1–3], the 5-year relative survival rate for the distant disease remains at 12% [4]. ccRCC is a highly vascularized tumor owing to an underlying mutation in the von-Hippel Lindau (*VHL*) gene, which leads to upregulation of VEGF. Cabozantinib, a multi-tyrosine kinase inhibitor (TKI), with targets including the VEGF receptor, inhibits angiogenesis and is one of the first-line single agent therapies approved for advanced ccRCC. Although it has shown improved survival compared to other first-line therapies [2, 5], many patients do not respond and will eventually progress [6]. Currently, there are no clinically approved biomarkers to identify patients with ccRCC who would be responsive or resistant to cabozantinib. Understanding why some patients develop resistance will be a key to developing tools to predict responders from non-responders and to identify different therapeutic targets.

Although not much is known regarding the immune-mediated mechanism of cabozantinib resistance, based on the studies on other VEGF inhibitors, one of the potential mechanisms of resistance is the compensatory upregulation of pro-angiogenic factors by immune cells in response to VEGFR blockade [7]. However, there are very limited data on how cabozantinib alters the immune environment in ccRCC. One preclinical study showed that cabozantinib treatment promoted the tumor-suppressing immune environment by increasing CD8+ T cells and by decreasing immunosuppressive regulatory T cells (Tregs) and myeloid-derived suppressor cells (MDSCs) [8]. A clinical study showed a decrease in Tregs in patients with metastatic urothelial cancer and a decrease in MDSCs in patients with triple negative breast cancer. Based on these findings, we focused our investigation on T cells and MDSCs as they can both promote and suppress tumor-associated angiogenesis [9–12]. Other studies have shown that anti-angiogenic therapies normalize blood vessel formation, facilitate trafficking of tumor-suppressive cells, and potentiate the efficacy of immunotherapy and cancer vaccines [13–15]. These studies have been the rationale for the combination of cabozantinib and immunotherapy, including a combination of cabozantinib with PD1/PD-L1 blocking antibodies [16–20]. Despite these therapeutic potentials of cabozantinib, some patients fail to respond to the treatment initially or develop resistance [21]. Therefore, it is important to elucidate how cabozantinib modulates immune cells and how immune cells may play a role in cabozantinib resistance.

In this study, we present a high throughput tri-culture model that incorporates cancer cells, endothelial cells, and patient-derived immune cells to study the effect of immune cells from patients with ccRCC on angiogenesis and cabozantinib resistance. We hypothesized that cabozantinib treatment upregulates non-VEGF pro-angiogenic factors and cytokines, which results in increased angiogenesis despite VEGFR inhibition. We show that circulating immune cells from patients with ccRCC induce cabozantinib resistance via increased secretion of a set of pro-angiogenic factors. Using multivariate partial least square regression (PLSR) modeling, we identified CD4+ T cell subsets that are correlated with cabozantinib resistance and report the changes in the frequency of these populations in ccRCC patients who are undergoing cabozantinib therapy.

## MATERIALS AND METHODS

### PBMC isolation

The research protocol to obtain whole blood from healthy donors and patients with ccRCC was approved by the Institutional Review Board at the University of Wisconsin. Informed consent was obtained prior to enrolling in the study. Blood was obtained from four healthy donors and seven patients with ccRCC. Blood samples were collected from ccRCC patients at a 4-week interval. Peripheral blood mononuclear cells were isolated using a density gradient-based separation method from SepMate™ (Stemcell) [22]. Briefly, freshly drawn whole blood at room temperature was diluted 1:1 with sterile DPBS. Then, 15 ml of Lymphoprep™ was added to a 50 ml SepMate™ tube, and 10–30 ml of diluted blood was layered on top of the Lymphoprep™. After 10-minute centrifugation at 1200 × *g* at room temperature, the top layer containing PBMCs was poured into a new centrifuge tube and was washed twice with sterile DPBS. Red blood cells were lysed using the RBC lysis buffer (Tonbo Biosciences). One to two million freshly isolated PBMCs were aliquoted for flow cytometry analysis of MDSCs to avoid decreases in the viability of granulocytic MDSCs following cryopreservation as previously reported [23]. The remaining PBMCs were frozen in freezing media (20% FBS + 10% DMSO in RPMI) and were stored in liquid nitrogen for lateruse.

### Cell cultures

Single-donor human umbilical vein endothelial cells (HUVECs) were purchased from Lonza (Cat. C2517A) and were cultured in EBM-2 media supplemented with EGMTM-2 Endothelial SingleQuotsTM Kit (Cat. CC-3162). Only cells between passages 4 and 5 were used for the experiment. 786O ccRCC cancer cell line was purchased from ATCC (ATCC, Cat. CRL-1932™) and were cultured in RPMI-1640 (ThermoFisher Scientific, Cat. 11875119) media supplemented with 10% fetal bovine serum (VWR) and 100 U/ml penicillin/streptomycin (ThermoFisher Scientific). Cultures were kept in a humidified incubator containing 5% CO_2_ at 37°C.

### Tri-culture and HUVEC tube formation assay

Tri-culture among cancer cells, endothelial cells, and PBMCs were performed using 384-well microDUO plates (Onexio Biosystems, Madison, WI) [24]. The microDUO plate allows the formation of a liquid bridge between adjacent wells by raising the media volume in the wells, connecting the cells populations cultured in each well via soluble factors. To incorporate cancer cells, 786O cells were trypsinized and 6000 cells in 20 μl were seeded onto the microDUO plate in alternating columns and were allowed to adhere for 6–8 hours. 786O media was then removed and was replaced with 20 μl of HUVEC media. Wells without 786O cells were coated with15 μl of growth factor-reduced, phenol-red free, LDEV-free Matrigel (Corning, #356231). The plate was centrifuged at 300 g for 5 minutes to remove any bubbles and was placed in a 37°C incubator for Matrigel to polymerize for 30 minutes. During this time, HUVECs were released using pre-warmed TrypLE Express (ThermoFisher). To minimize clumping, HUVECs were incubated with 20 mg/ml DNase for 20 minutes. After the Matrigel had solidified, 6000 HUVECs in 5 μl (1.2 × 10^6^ cells/ml) were added on top of the Matrigel and were incubated for 1 hour. Then, 30 000 PBMCs in 20 μl were added to the wells containing 786O ccRCC cells and were centrifuged briefly at 150 g to pull down the PBMCs. Additional 20 μl HUVEC media with or without cabozantinib was added to each well gently to establish the media bridge, and the tri-culture was maintained for 16–18 hours.

### Inhibitor treatment

Cabozantinib (Selleckchem, Cat. S1119) was diluted in DMSO (Millipore Sigma, Cat.D2650) to a stock concentration of 20 mM. When indicated, cabozantinib was diluted in the media to a final concentration of 10 μM and was added to the culture. DMSO was used for the control conditions.

### Quantification of tube formation assay

After 16–18 hours of tri-culture, the plate was removed from the incubator, and phase-contrast images of each well were obtained using a Nikon TI® Eclipse inverted microscope (Melville, NY) with NIS Element. The total length of the tubes formed was quantified using the Angiogenesis Analyzer plugin in ImageJ [25].

### Secreted protein measurements

After 16–18 hours of tri-culture in the microDUO plates, conditioned media was collected from both wells and were centrifuged at 300 g for 5 minutes in 4°C to remove cells. The supernatant was collected and was transferred to a microcentrifuge tube where it was stored at −80°C until use. The secreted protein measurements were performed using the Magnetic Bead-Based Multiplex ELISA system MAGPIX (Luminex Corporation) with a custom-made panel (R&D Systems) according to the manufacturer’s protocol. The custom-made panel was used to quantify the following analytes: IL1b, IL-6, IL-8, IL-10, IL12/23, metalloproteinase 9 (MMP9), heparin binding-EGF (HB-EGF), PDGF-BB, angiopoietin-2, VEGF, VEGF-C, Gro-a, Leptin, G-CSF, Tie-2, C-C motif chemokine ligand 2 (CCL2), CCL11, HGF, BMP-9, and uPA (R&D Systems). Data were collected with xPonent software (Luminex), and soluble factor concentrations in media were calculated using mean fluorescence intensities by creating a standard curve for each analyte using a five-parameter logistic curve fit in Graphpad Prism8 (GraphPad Software, La Jolla,CA).

### Flow cytometry

MDSCs were profiled immediately after isolation from the fresh whole blood. T cells were profiled after cryopreservation. The following panel was used to profile T cells: CD3-PEcy5, CD4-BUV805, CD4-BB700, CD45RA-BUV563, CCR7 Alexa 700, CXCR5-BV480, CCR6-BV421, CCR4-BV605, CXCR3-PE, CCR10-APC, CD45RO-PE-Cy7, and PD1-BUV395; and the following panel was used to profile MDSCs: CD14-PE-CF594, CD11b-BV605, HLA-DR-BV421, Lineage markers-APC, CD33-PE, and CD15-PECy7. A detailed list of antibodies and markers used to identify each cell type is given in Supplementary Table 1. PBMCs were collected, centrifuged, and resuspended in DPBS. All cells were stained using Ghost Red 780 (Tonbo Biosciences) for 30 minutes on ice according to the manufacturer’s protocol. Cells were washed with DPBS and resuspended in Brilliant Staining Buffer (BD Bioscience) and were incubated for 10 minutes with Fc Block (BD Bioscience). Following the incubation, appropriate antibodies were added and incubated for 30 minutes in the dark at room temperature. After the incubation, cells were washed using the running buffer (2 mM EDTA, 0.5% BSA in sterile DPBS) and were resuspended in the running buffer. Fluorescence minus one controls were prepared for all antibodies. Compensation samples were prepared using UltraComp eBeads™ (ThermoFisher, Cat. 01-2222-42). The gating strategy used to identify T cell subsets is shown in Supplementary Fig. 1 [26] and the gating strategy used to identify MDSC subset is shown in Supplementary Fig. 2 [27]. Briefly, cells were first gated based on size to exclude debris and then gated on singlets followed by live-cell gating to exclude dead cells. For T cells, all CD3+ cells were analyzed. Cells were analyzed using BD FACSAria III cell sorter, and the data were analyzed using the FLOWJO (Tree Star, Ashland, OR) software.

### PLSR analysis

MxN data matrix was generated with data from M patients and N MDSC and T cell flow cytometry signals. Each column of the independent X matrix corresponds to a unique input or signal: individual MDSC and T cell subtypes and each column of the dependent Y matrix correspond to unique cabozantinib resistance quantified by total tube length after cabozantinib treatment in the tri-culture model. Each row represents a unique patient. All data were mean-centered and were scaled to unit variance. SIMCA-P (UMetrics) was used to solve the PLSR problem with the nonlinear iterative partial least squares algorithm [28].

### mRNA signature analysis

The Cancer Genome Atlas (TCGA) ccRCC primary tumor RNA-seq gene expression data (in log_2_(TPM + 1)) were downloaded from UCSC Treehouse (https://xena.treehouse.gi.ucsc.edu/download/TumorCompendium_v10_PolyA_hugo_log2tpm_58581genes_2019-07-25.tsv). Patient clinical data were downloaded from Broad Institute (http://gdac.broadinstitute.org). Five hundred and thirty ccRCC patients have both primary tumor RNA-seq and clinical data available. Expression of type 9 helper T cells (Th9) and IL-22 secreting helper T cells (Th22) gene signatures (Supplementary Table 2) were defined as the average of its genes’ expression levels. Patients were stratified by the median expression levels of gene signatures and were equally divided into high and low expression groups with 265 patients in each group. A log-rank test was used to test the significance of correlation with overall survival.

### Statistical analysis

Angiogenesis experiments were performed using immune cells isolated from seven different patients with ccRCC and from four different healthy donors. All results are presented as the mean ± standard deviation (SD). Data were analyzed using GraphPad Prism v8. Statistical significance was set at *P* < 0.05. One-to-one comparisons were performed with a Student’s *t*-test. A Welch’s correction was performed for the analytes with unequal variance. The heatmap was generated using Broad Institute’s Morpheus (https://software.broadinstitute.org/morpheus).

## RESULTS

### Immune cells from patients with ccRCC confer resistance to cabozantinib treatment

**Figure 1:**
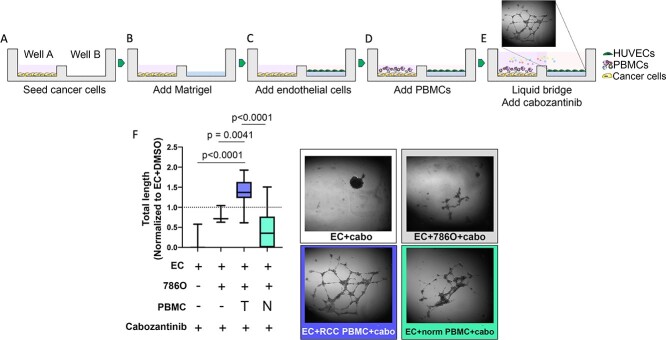
PBMCs from ccRCC patients confer resistance to cabozantinib treatment. (A–E) Schematic of the experimental set-up. microDUO, high throughput 384-well open microchannel multi-culture plates (Onexio Biosystems) were used to determine the effect of ccRCC PBMCs on angiogenesis. (F) The total length of the endothelial tubes was normalized to EC + DMSO-only control condition (dotted line).

Both adaptive and innate immune cells have shown to be important in overcoming VEGF inhibition by TKIs—in particular, sunitinib, another TKI approved to treat advanced ccRCC [29, 30]. However, not much is known about whether immune cells play a role in cabozantinib resistance. Although they both block VEGF receptor 2, which is the main signaling VEGF receptor on vascular endothelial cells [31], Cabozantinib also blocks MET and AXL [32], whereas sunitinib blocks VEGFR1–3, platelet-derived growth factor (PDGF), and c-kit receptor [33]. Therefore, to determine whether the immune cells from ccRCC patients influence the effect of cabozantinib on angiogenesis, we set up a high throughput tri-culture angiogenesis assay using 384-well microDUO plates (Onexio Biosystems) [24]. The unique design of these plates allows different cell types to be cultured in individual wells in a small media volume. When this volume is increased, a media bridge is formed connecting adjacent wells, allowing cell-to-cell communication via soluble factors through the media bridge without direct cell-to-cell interactions. Using this plate, we first seeded 786O RCC cell line into well A (Fig. 1A). 786O RCC cells were chosen because they were shown to resemble ccRCC including the underlying VHL mutation [34, 35]. After 6–8 hours allowing the cancer cells to adhere, we then coated well B with a thick layer of Matrigel (Fig. 1B). After the Matrigel solidified, HUVECs were overlaid on the Matrigel in well B and were cultured for 1 hour to allow the HUVEC to adhere to the Matrigel (Fig. 1C). Next, PBMCs were added to well A and were briefly centrifuged to pull down the PBMCs (Fig. 1D). Lastly, media with or without 10 μM (final concentration) cabozantinib was added to both wells carefully to bridge the two wells with a media bridge, establishing the tri-culture with soluble communication between the wells (Fig. 1E). After 14–16 hours, tube formation was imaged using an inverted microscope and was quantified using the Angiogenesis Analyzer plugin in ImageJ [25]. The total length of the endothelial tubes was normalized to endothelial cells + DMSO control. In the absence of cancer cells or immune cells, cabozantinib decreased endothelial tube formation by 81% when compared to endothelial cells without cabozantinib (Fig. 1F). In the presence of the 786O cancer cells, the tube formation was reduced by only 20%. However, the addition of PBMCs from patients with ccRCC to the co-culture mitigated this effect and actually increased tube formation (40%) in response to cabozantinib treatment (*P* = 0.0041), but there was no increase with healthy donor PBMCs (*P* = 0.27). To determine whether ccRCC PBMC-mediated cabozantinib resistance was specific to the clear cell phenotype of 786O cells, the experiment was repeated using A498 cells, another RCC cell line that resembles a papillary phenotype [36, 37] and has differential gene expression and cytokine profiles. [35, 38] Cabozantinib resistance was not seen with A498 cells (Supplementary Fig. 4). These findings suggest that the immune cells from patients with ccRCC can confer resistance to cabozantinib and increase angiogenesis in ccRCC.

**Figure 2:**
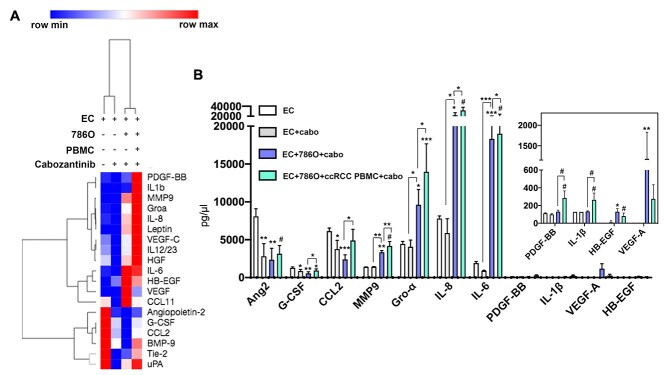
PBMCs from patients with ccRCC upregulated secretion of pro-angiogenic factors in response to cabozantinib treatment. (A) The concentration of pro-angiogenic factors was quantified using a multiplex magnetic bead-based assay (R&D Systems). The heatmap was used to visualize the differential regulation of pro-angiogenic factors in response to cabozantinib treatment in the absence and presence of cancer cells and ccRCC PBMCs. (B) The concentration of pro-angiogenic cytokines and growth factors obtained from conditioned media from mono-culture, co-culture, and tri-culture conditions treated with cabozantinib. Bars represent average ± SD of *n* = 3 wells for EC + DMSO, EC + cabozantinib, EC + 786O + cabozantinib, and *n* = 9 wells (three wells per patient, three patients in total) (^*^P ≤ 0.05, ^**^P ≤ 0.01, ^***^P ≤ 0.005, and #*P* ≤ 0.0001).

**Figure 3:**
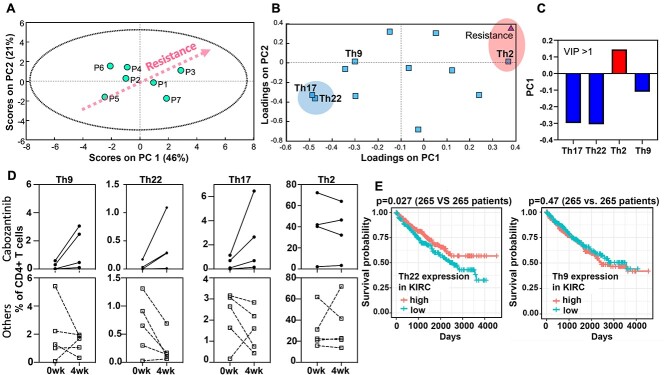
PLSR analysis of MDSC and T cell subsets and cabozantinib resistance. PBMCs isolated from nine patients with ccRCC undergoing systemic therapy were profiled for MDSC and T cell subtypes using flow cytometry. A PLSR model was generated using flow cytometry data as independent variables and cabozantinib resistance quantified by total tube length from tri-culture angiogenesis assay as a dependent variable. (A) Scores plot separated patients according to the degree of resistance measured in the tri-culture model along the principal component 1 (PC1). (B) Loadings plot shows covariance among the immune cell subtypes with the cabozantinib resistance. (C) VIP of immune cell subtypes with VIP score >1. (D) Changes in Th9, Th17, Th22, and Th2 cells for four patients who are receiving cabozantinib treatment (solid lines) were compared to five patients who are undergoing other therapies (dotted lines, three patients for nivolumab and two patients for pazopanib). (E) Immune gene-signature analysis of 530 patients with ccRCC from TCGA cohort showed that patients with higher expressions of the Th22 gene signature had significantly better overall survival (log-rank test *P* = 0.027). There was no significant correlation between the Th9 gene signature and overall survival.

### Upregulation of pro-angiogenic factors by RCC immune cells leads to cabozantinib resistance

Blocking VEGF has been shown to lead to a compensatory increase in other pro-angiogenic factors by tumor leading to resistance. [36] Therefore, we hypothesized that the ccRCC PBMCs confer cabozantinib resistance by secretion of non-VEGF pro-angiogenic proteins. After imaging the tube formations, we collected the conditioned media from each tri-culture well and measured 19 secreted pro-angiogenic proteins using a multiplex magnetic bead-based immunoassay (R&D Systems). The results are visualized as a heatmap to show an overview of differential regulation of pro-angiogenic factors upon cabozantinib treatment in the absence and presence of cancer cells and ccRCC PBMCs (Fig. 2A)—when endothelial cells were treated with cabozantinib, there was a clear decrease in a number of pro-angiogenic factors, but the addition of cancer cells and ccRCC PBMCs resulted in a persistent elevation of multiple pro-angiogenic factors. Specifically, cabozantinib inhibited secretion of angiopoietin-2, G-CSF, interleukin- (IL-6), and CCL2 by endothelial cells (Fig. 2B). The addition of 786O cancer cells led to an increase in MMP9, Gro-**α**, IL-8, IL-6, HB-EGF, and VEGF-A; and the addition of ccRCC PBMCs led to an increase in PDGF-BB and IL-1β as well as a further increase in MMP-9, Gro-**α**, IL-8, IL-6, and CCL-2 despite cabozantinib treatment. (Fig. 2B). These results support our hypothesis that ccRCC PBMC confers cabozantinib resistance through persistent secretion of non-VEGF pro-angiogenic factors.

### Lower Th17, Th22, and Th9 cell populations are associated with cabozantinib resistance

Cabozantinib was shown to increase CD4+ and CD8+ T cells and reduce the number of splenic Tregs and MDSCs in a preclinical study [8]. However, not much is known about how cabozantinib affects immune cells in patients with ccRCC. Therefore, we sought to determine the effect of cabozantinib treatment on T cell and MDSC populations. Specifically, we wanted to determine if there is any correlation between changes in the immune population and cabozantinib resistance and sensitivity. To test this, we profiled the T cell subsets and MDSCs in PBMCs isolated from ccRCC patients who were receiving cabozantinib as well as from patients who were receiving other systemic therapies. Specifically, the T cell subset profiles tested consisted of types 1, 2, 9, 17, and 22 helper T cells, follicular-like helper T cells, GM-CSF secreting CD4+ T cells, Tregs, PD1+ CD8 T cells, and PD1+ CD4 T cells [26]. The MDSCs’ subset profiles consisted of monocytic MDSCs, granulocytic MDSCs, and early-stage MDSCs [27].

Then, we created a computational model based on PLSR analysis to determine if there was a mathematical correlation between immune cell subtypes (MDSCs and T cells) and cabozantinib resistance as quantified by microDUO endothelial tube formation assay. PLSR modeling uses a linear combination of variables (immune cell subset in this case) reduced into a smaller dimension of principal components (PCs) to explain the variance in the dependent variable (cabozantinib resistance). Loadings can be calculated for each variable to determine which variables can best explain the dependent variable. Highest loadings represent independent variables that co-vary the most with the dependent variable [37]. In our model, the first two PCs captured 46% and 21% variability found in the dataset (Fig. 3A). Patient 3 with the highest cabozantinib resistance *in vitro* were at the right upper quadrant. The loading plot (Fig. 3B) shows how each immune cell subtype contributes to the cabozantinib resistance, where cells that are nearest to the point labeled ‘resistance’ contribute positively to the resistance and the cells that are farthest contribute negatively to cabozantinib resistance. Next, we calculated the variable importance in projection (VIP) score that quantifies the variables (immune cells) that are important for cabozantinib resistance (Fig. 3C). VIP score >1 is considered to be significant. The model suggested that Th9, Th22, and Th17 cells contribute negatively to cabozantinib resistance and that Th2 cells contribute positively to cabozantinib resistance. In other words, lower levels of Th9, Th22, and Th17 cells and higher levels of Th2 cells are associated with greater cabozantinib resistance. However, the number of PD1+ CD8+ and PD1+ CD4+ cells did not contribute significantly to cabozantinib resistance in the model. Because there have been no reported studies investigating these T cell subtypes and cabozantinib resistance, we investigated the changes of the T cell subtypes (i.e. Th9, Th22, Th17, and Th2) over a 1-month period from four ccRCC patients who were receiving cabozantinib treatment and from five patients who were undergoing other systemic therapies (i.e. three patients receiving nivolumab and two patients receiving pazopanib). Based on the PLSR model, we expected to see higher levels of Th9, Th22, and Th17 and a decrease in Th2 in the patients receiving cabozantinib treatments. To test this, blood was obtained 1 month apart at two different time points, and PBMCs were isolated. Then, T cells and MDSCs were profiled using flow cytometry. The underlying assumption here was that the patients continued successful therapy with cabozantinib within the 1-month time interval without experiencing treatment resistance. Interestingly, we observed an increase in Th9, Th22, and Th17 cells from those patients who continued to receive cabozantinib treatment (Fig. 3D, solid lines) but not from the patients who were receiving different therapies (Fig. 3D, dotted lines). Changes in Th2 cells were inconsistent among the patients receiving cabozantinib. There was no change in Th1, Treg, and CD8+ T cell populations (Supplementary Fig. 3). Previous study on The Cancer Genome Atlas (TCGA) [38] ccRCC patients had shown that patients with higher expressions of Th17 gene signature had significantly better overall survival [39]. We decided to examine if expression levels of Th9 and Th22 gene signatures (Supplementary Table 3) also correlated with overall survival. In our analysis of RNA-seq data from 530 TCGA ccRCC patients, we found that higher expression of Th22, but not Th9, the signature had significantly better overall survival (Fig. 3E). These findings suggest that the changes in these T cell subsets can be useful in determining whether a patient will respond to cabozantinib and can have implications in survival.

## DISCUSSION

The goal of this study was to elucidate the effect of circulating immune cells on angiogenesis in response to cabozantinib treatment when other factors are kept constant. Here, we showed that immune cells from patients with ccRCC confer resistance to cabozantinib treatment by upregulating pro-angiogenic factors when VEGF-A and HB-EGF were suppressed. Using multivariate analysis of immune subsets, we identified three helper T cell subsets (Th9, Th22, and Th17) that are correlated with cabozantinib resistance.

Inhibition of pro-angiogenic tyrosine kinases leads to compensatory increases in other pro-angiogenic factors from the tumor microenvironment [36]. In our study, secretion of IL-6, IL-8, MMP-9, PDGF-BB, IL-1β, G-CSF, and CCL-2 by ccRCC immune cells in tumor microenvironment were able to overcome the anti-angiogenic effects of cabozantinib. Although there are no clinical data available on serum- or tumor tissue-level expression of these pro-angiogenic factors for ccRCC patients who are resistant to cabozantinib treatment, a number of studies have shown upregulation of these factors in advanced ccRCC. Specifically, higher levels of IL-6 have been associated with more aggressive ccRCC and worse survival [40–42]. Further, increased IL-6 levels after TKI treatment and subsequent inhibition of the IL-6 signaling pathway enhanced efficacy of sorafenib, another TKI that blocks VEGF and PDGF receptors, which is approved for advanced RCC [43] on suppressing angiogenesis and tumor growth [44]. In an exploratory analysis of a clinical trial comparing cabozantinib and everolimus, a different kinase inhibitor, for the treatment of advanced ccRCC, low baseline plasma levels of VEGF and IL-8 were prognostic for better overall survival among patients receiving cabozantinib [45]. In addition, a higher baseline level of MMP-9 has been shown in those patients with metastatic RCC who did not respond to sunitinib [46]. As for IL1β, it promotes ccRCC cell invasion by upregulating secretion of MMPs [47] and promotes angiogenesis by stimulation of VEGF secretion [48, 49]. Additionally, CCL2 [50] has been found to recruit tumor associated macrophages, which are known to confer resistance to anti-VEGF therapies by participating in vascular sprouting [51]. Lastly, activation of the PDGF/PDGFR pathway has been suggested as a potential escape mechanism for VEGFR blockade [52] and is likely to play a role in cabozantinib resistance. These studies support our finding that compensatory upregulation of pro-angiogenic factors in ccRCC patients receiving cabozantinib treatment as one potential mechanism of treatment resistance. Furthermore, there are currently no clinically approved biomarkers to identify patients who may be responsive or resistant to cabozantinib. The secreted pro-angiogenic factors identified in this study are potential biomarkers that could be studied in larger clinical studies for personalized medicine.

For this study, we used PBMCs as a surrogate for tumor-infiltrating immune cells. PBMCs were chosen not only for their ease of acquisition but also for their potential as circulating biomarkers that are readily accessible in clinical settings and would allow all patients to be investigated, not just those that would receive surgery or biopsies. Although PBMCs do not interact directly with cancer cells and may have differences in subsets and sensitization from the distinct intratumoral immune cell populations, there is evidence that certain T cell clonotypes found in the tumor are found in both the adjacent normal tissue and peripheral blood, suggesting ongoing replenishment of certain tumor T cell population by peripheral cells [53].

Adaptive immune cells, along with innate immune cells, have been shown to play roles in overcoming VEGF inhibition by promoting tumor angiogenesis in RCC and other solid tumors. Here, we identified three helper T cell subsets (Th9, Th22, and Th17) that are correlated with cabozantinib resistance. Although there are limited studies on the effect of cabozantinib on T cell polarization in ccRCC, an association between various T cell subtypes and tumor grade and disease progression has been gathered in clinical studies. Th17 cells have been shown to induce resistance to VEGF inhibition by promoting secretion of G-CSF and the recruitment of immature myeloid cells to support tumor angiogenesis [54]. A study analyzing gene expression of 488 patients with ccRCC correlated higher Th17 cell gene expression with better survival [39]. On the other hand, Th9 and Th22 [55] are relatively rare T cell subtypes in the context of solid tumors; therefore, the information on these two cell types in ccRCC is very limited. In our analysis of TCGA gene expression data on patients with ccRCC, we saw that higher Th22 gene expression was associated with better survival. Interestingly, Th22 can exert either pro-inflammatory or immunosuppressive effects depending on the context [56]. In a xenograft model of RCC using the A498 cell line, IL-22, a cytokine secreted by Th22 cells, slowed the tumor growth [57]. Accumulation of Th22 cells has been correlated with poor progression in other cancer types including gastric cancer [58] as well as patients with late stage and refractory multiple myeloma [59]. As for Th9 cells, they have been shown to eradicate advanced tumors in a murine model of melanoma [60]. Although a preclinical study showed an increase in peripheral CD8+ T cells and decrease in Tregs after cabozantinib treatment [8], we did not observe consistent changes in these populations among the four patients who were treated with cabozantinib (Supplementary Fig. 3). Our findings suggest that Th9, Th22, and Th17 cells could be useful in determining cabozantinib sensitivity and survival, but longitudinal analysis in a larger cohort of patients receiving cabozantinib will be needed to determine its clinical utility.

It is challenging to study patient-specific immune effects on TKI resistance in clinical studies and in patient-derived xenograft models; therefore, there is a need for improved *in vitro* models. There are limitations in our approach, namely, that our model lacks a fully 3D extracellular matrix and luminal configuration of endothelial tubes, which could potentially affect cell phenotype [61]. Additionally, in its current configuration, the endothelial cells do not make direct cell: cell contacts with epithelial cells and PBMC. However, the system can be easily modified to study the effects of direct physical interactions instead of crosstalk by soluble factors only. Despite these limitations, the multi-culture system used in this study offers numerous advantages, including isolating the effect of patient-derived immune cells on anti-angiogenic therapy. Another advantage of this model is that it can be applied to study interactions among other cells (e.g. endothelial, epithelial, fibroblast, and immune) isolated from a single patient. Finally, the model could be used to screen multiple anti-angiogenic therapies as well as combination therapies with immunotherapies.

In summary, we showed a potential resistance mechanism for cabozantinib resistance mediated by immune cells and identified rare T cell immune subsets that are associated with cabozantinib resistance. These findings should be further investigated among the patients with ccRCC receiving cabozantinib in clinical trials and as part of the standard of care to validate their clinical utility in predicting the patients who would benefit from cabozantinib treatment.

## Disclosures

D.J.B. holds equity in Bellbrook Labs LLC, Tasso Inc., Salus Discovery LLC, Lynx Biosciences Inc., Stacks to the Future LLC, Turba LLC, Flambeau Diagnostics LLC, and Onexio Biosystems LLC. D.J.B. is also a consultant for Abbott Laboratories.

## Authors’ contributions

K.P. conceived the method and designed the research. K.P. and H.O.H. performed the experiments with assistance from K.M.L.-C. and S.C.K K.P. and P. L. analyzed the raw data and prepared data visualization with assistance from K.M.L.-C. and S.C.K D.J.B. supervised experimental design, data analysis, and data presentation. K.P. and D.J.B. wrote the manuscript, and all authors revisedit.

## Supplementary Material

Supplementary_figure_1_zyab018

Supplementary_Figure_2_zyab018

Supplementary_Figure_3_zyab018

Supplementary_Figure_4_zyab018

Supplementary_table_1_zyab018

Supplemental_table_2_zyab018

Supplementary_Table_3_zyab018
