# Supplementary figures and images for: Immune cell mediated cabozantinib resistance for patients with renal cell carcinoma
Source: Integr Biol (Camb). 2021 Dec 21;13(11):259–68. doi: 10.1093/intbio/zyab018 (PMC8730366; doi:10.1093/intbio/zyab018)

Supplementary figure 1. Flow cytometry gating strategy for T-cells


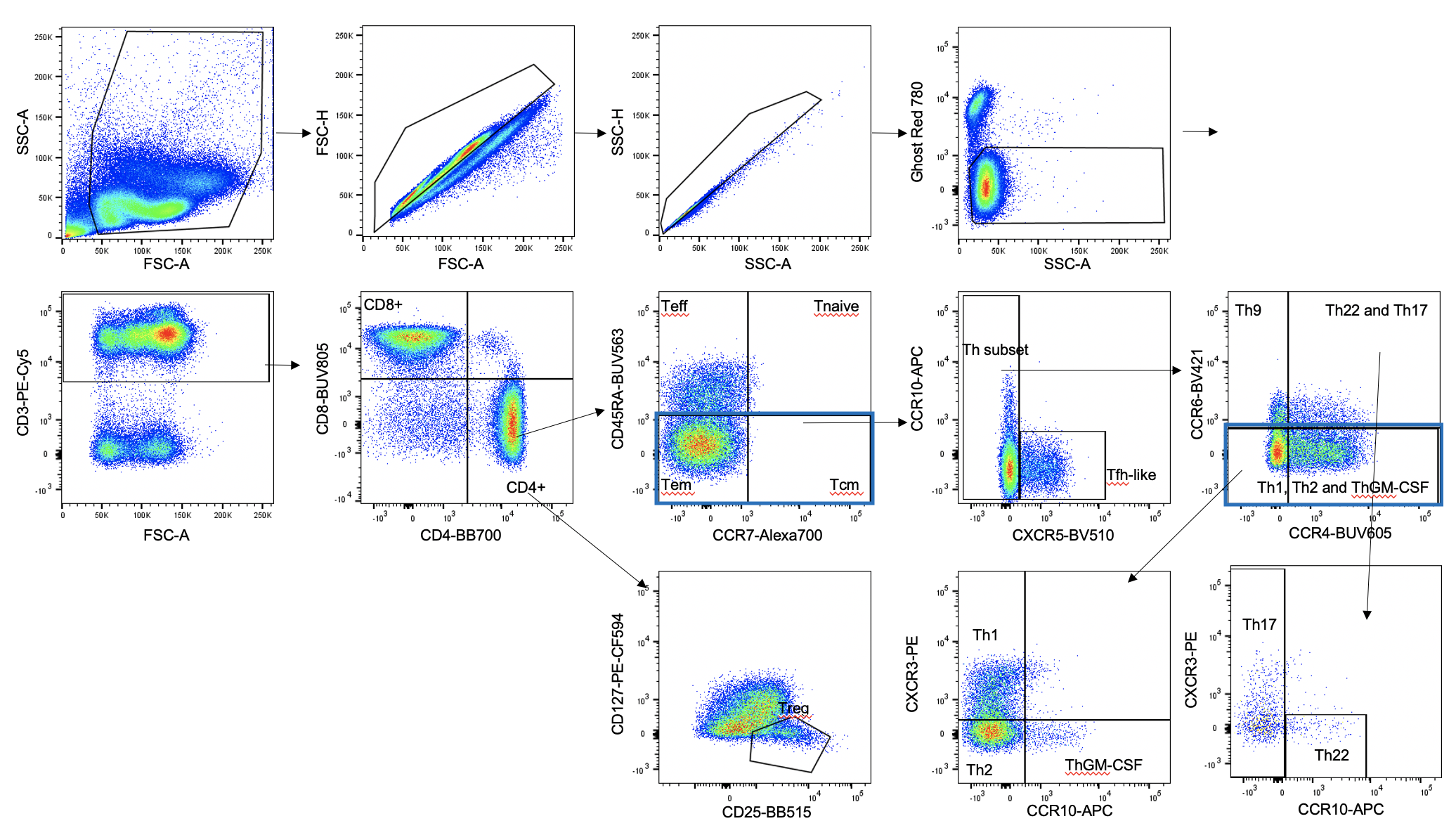

Supplement: Supplementary_figure_1_zyab018 [file supplementary_figure_1_zyab018.docx]
