# Supplementary material for: Immune cell mediated cabozantinib resistance for patients with renal cell carcinoma
Source: Integr Biol (Camb). 2021 Dec 21;13(11):259–68. doi: 10.1093/intbio/zyab018 (PMC8730366; doi:10.1093/intbio/zyab018)

Supplementary Figure 2. Flow cytometry gating strategy for myeloid derived suppressor cells (MDSCs)


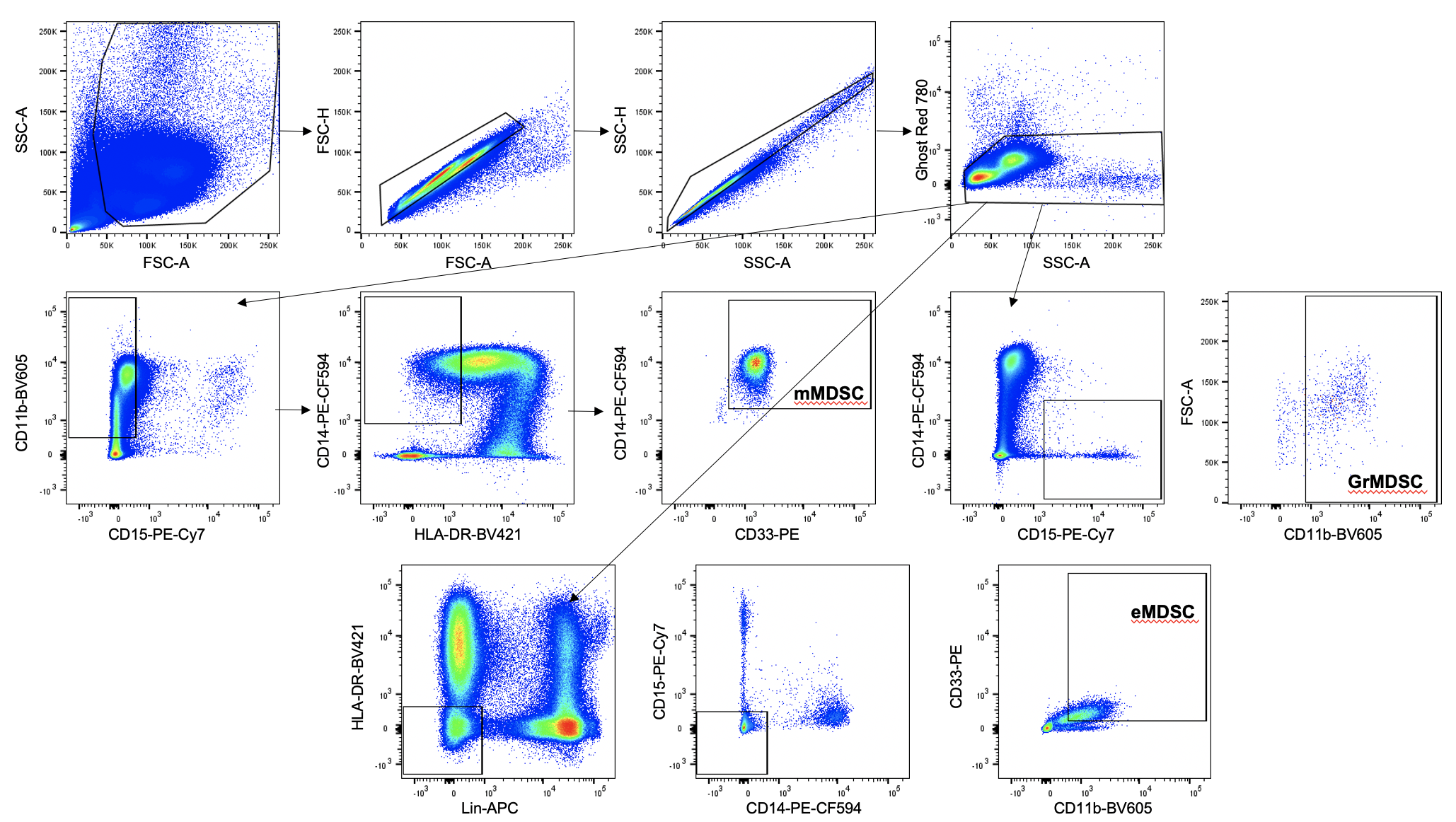

Supplement: Supplementary_Figure_2_zyab018 [file supplementary_figure_2_zyab018.docx]
