# Supplementary material for: Immune cell mediated cabozantinib resistance for patients with renal cell carcinoma
Source: Integr Biol (Camb). 2021 Dec 21;13(11):259–68. doi: 10.1093/intbio/zyab018 (PMC8730366; doi:10.1093/intbio/zyab018)
Supplement: Supplementary_Figure_3_zyab018 [file supplementary_figure_3_zyab018.docx]

**Supplementary Figure 3.** Changes in regulatory T cells, CD8+ T cells and CD4+ Th1 cells for patients with ccRCC receiving cabozantinib and other systemic therapies.

**
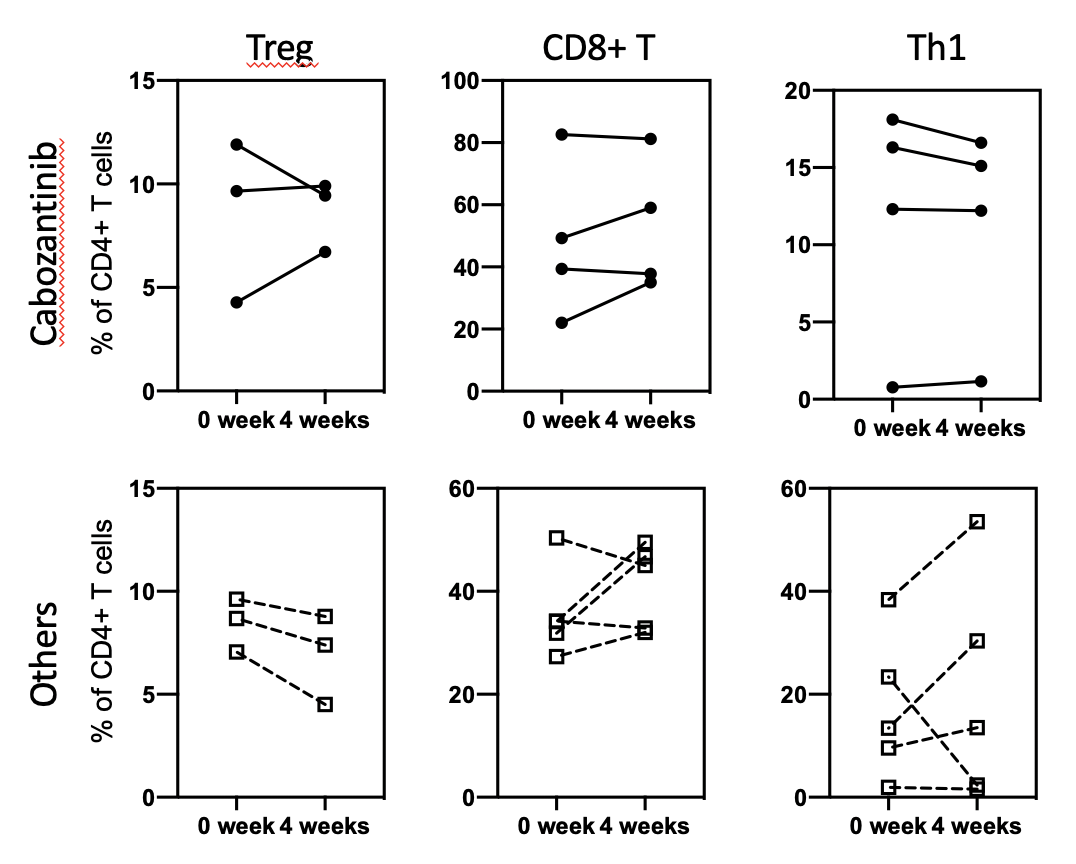
**
