# Supplementary material for: Immune cell mediated cabozantinib resistance for patients with renal cell carcinoma
Source: Integr Biol (Camb). 2021 Dec 21;13(11):259–68. doi: 10.1093/intbio/zyab018 (PMC8730366; doi:10.1093/intbio/zyab018)
Supplement: Supplementary_Figure_4_zyab018 [file supplementary_figure_4_zyab018.docx]

**Supplementary Figure 4.** When co-cultured with different ccRCC cell line A498, PBMCs from ccRCC patients did not confer resistance to cabozantinib treatment

**
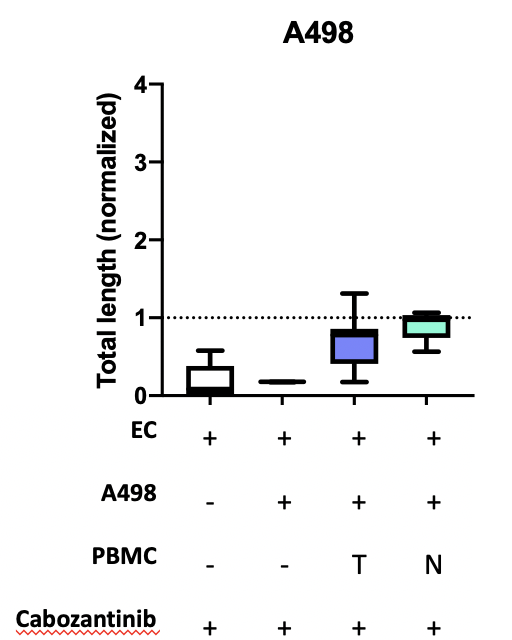
**
