# Supplementary material for: Immune cell mediated cabozantinib resistance for patients with renal cell carcinoma
Source: Integr Biol (Camb). 2021 Dec 21;13(11):259–68. doi: 10.1093/intbio/zyab018 (PMC8730366; doi:10.1093/intbio/zyab018)
Supplement: Supplementary_table_1_zyab018 [file supplementary_table_1_zyab018.docx]

Supplementary table 1. The antibody panel used to detect T cell subtypes and myeloid-derived suppressor cells

| Marker | | Fluorochrome | Clone | Source | Catalogue# | Dilution |  |
| --- | --- | --- | --- | --- | --- | --- | --- |
| T cell panel | | | | | | |  |
| CD3 | | PE-Cy5 | UCHT1 | Tonbo Biosciences | 55-0038 | 1:20 |  |
| CD8 | | BUV805 | SK1 | BD Biosciences | 564913 | 1:20 |  |
| CD4 | | BB700 | SK3 | BD Biosciences | 566393 | 1:20 |  |
| CD45RA | | BUV563 | HI100 | BD Biosciences | 565703 | 1:20 |  |
| CCR7 | | Alexa 700 | 150503 | R&D | FAB197N-025 | 1:20 |  |
| CD185 (CXCR 5) | | BV480 | RF8B2 | BD Biosciences | 566191 | 1:20 |  |
| CCR6 (CD 196) | | BV421 | G034E3 | BioLegend | 353407 | 1:20 |  |
| CD194 (CCR4) | | BV605 | L291H4 | BioLegend | 359417 | 1:20 |  |
| CD183 (CXCR3) | | PE | 1C6 | BD Biosciences | 557185 | 1:5 |  |
| CCR10 | | APC | 314305 | R&D | FAB3478A-025 | 1:20 |  |
| CD25 | | BB515 | 2A3 | BD Biosciences | 564468 | 1:20 |  |
| CD127 | | PE-CF594 | HIL-7R-M21 | BD Biosciences | 562397 | 1:20 |  |
| PD-1 | | BUV395 | MIH4 | BD Biosciences | 745619 | 1:20 |  |
| CD45RO | | PE-Cy7 | UCHL1 | BioLegend | 304229 | 1:20 |  |
| Ghost Dye Red 780 | |  | N/A | Tonbo Biosciences | 3-0865-T100 | 1:1000 |  |
| MDSC Panel | | | | | | |  |
| CD14 | | PE-CF594 | MφP9 | BD Biosciences | 562334 | 1:20 |  |
| CD11b | | BV 605 | ICRF44 | BioLegend | 301331 | 1:20 |  |
| HLA DR | | BV421 | G46-6 | BD Biosciences | 562804 | 1:20 |  |
| Lineage Cocktail 1 (CD3/CD14/CD16/CD19/CD20/CD56) | | APC | OKT3 M5E2 3G8 HIB19 2H7 HCD56 | BioLegend | 348807 | 1:20 |  |
| CD33 | | PE | WM53 | BioLegend | 303404 | 1:20 |  |
| Ghost Dye Red 780 | |  | N/A | Tonbo Biosciences | 13-0865-T100 | 1:1000 |  |
| CD15 | | PE-Cy7 | W6D3 | Tonbo Biosciences | 323030 | 1:20 |  |
|  |  |  |  |  |  |  |  |
