# Supplementary material for: Immune cell mediated cabozantinib resistance for patients with renal cell carcinoma
Source: Integr Biol (Camb). 2021 Dec 21;13(11):259–68. doi: 10.1093/intbio/zyab018 (PMC8730366; doi:10.1093/intbio/zyab018)
Supplement: Supplemental_table_2_zyab018 [file supplemental_table_2_zyab018.docx]

Supplemental table 2. The gating strategies

| Cell type | Markers |
| --- | --- |
| Type 1 helper T cells (Th1) | CD4+CD45RA-CXCR5-CCR6-CXCR3+CCR10- |
| Type 2 helper T cells (Th2) | CD4+CD45RA-CXCR5-CCR6-CXCR3-CCR10- |
| Type 9 helper T cells (Th9) | CD4+CD45RA-CXCR5-CCR6+CCR4- |
| Type 17 helper T cells (Th17) | CD4+CD45RA-CXCR5-CCR6+CCR4-CCR10- |
| Type 22 helper T cells (Th22) | CD4+CD45RA-CXCR5-CCR6+CCR4-CCR10+ |
| GM-CSF helper T cells | CD4+CD45RA-CXCR5-CCR6-CXCR3-CCR10+ |
| Follicular-like helper T cells (Tfh-like) | CD4+CD45RA-CXCR5+ |
| CD45RO+PD1+ CD8+ T cells | CD8+CD45RO+PD1+ |
|  |  |
| Early-stage MDSC (eMDSC) | Lin-HLA-DR-CD14-CD15-CD33+CD11b+ |
| Monocytic MDSC (mMDSC) | CD11b+CD14+HLA-DRlow/- CD15- |
| Granulocytic MDSC (GrMDSC) | CD14-CD11b+CD15+ |
