# Supplementary material for: Immune cell mediated cabozantinib resistance for patients with renal cell carcinoma
Source: Integr Biol (Camb). 2021 Dec 21;13(11):259–68. doi: 10.1093/intbio/zyab018 (PMC8730366; doi:10.1093/intbio/zyab018)
Supplement: Supplementary_Table_3_zyab018 [file supplementary_table_3_zyab018.docx]

Supplementary Table 3. Immune gene signatures

| Name | Source | Gene count | Genes |
| --- | --- | --- | --- |
| Th9 |  | 7 | IL9, EOMES, GZMB, TNFSF14, TNFSF4, TNFSF8, and ICOS |
| Th22 |  | 15 | PDGFRA, PDGFRB, FGFR1, FGFR2, FGFR3, FGFR4, IL22, FGF1, FGF5, FGF12, FGF13, CCL7, CCL15, BNC2, and FOXO4 |
